# Circulating angiopoietin-like protein 8 (ANGPTL8) and steatotic liver disease related to metabolic dysfunction: an updated systematic review and meta-analysis

**DOI:** 10.3389/fendo.2025.1574842

**Published:** 2025-04-10

**Authors:** Farah Abdelhameed, Lukasz Lagojda, Chris Kite, Alexander Dallaway, Attia Mustafa, Nwe Ni Than, Eva Kassi, Harpal S. Randeva, Ioannis Kyrou

**Affiliations:** ^1^ Warwickshire Institute for the Study of Diabetes, Endocrinology and Metabolism (WISDEM), University Hospitals Coventry and Warwickshire NHS Trust, Coventry, United Kingdom; ^2^ Institute for Cardiometabolic Medicine, University Hospitals Coventry and Warwickshire NHS Trust, Coventry, United Kingdom; ^3^ Sheffield Centre for Health and Related Research (SCHARR), School of Medicine and Population Health, University of Sheffield, Sheffield, United Kingdom; ^4^ School of Health and Society, Faculty of Education, Health and Wellbeing, University of Wolverhampton, Wolverhampton, United Kingdom; ^5^ Faculty of Health, Medicine and Society, Division of Public Health, Sport and Wellbeing, University of Chester, Chester, United Kingdom; ^6^ Warwick Medical School, University of Warwick, Coventry, United Kingdom; ^7^ Internal Medicine Department, Faculty of Medicine, Omar Almukhtar University, Al-Bayda, Libya; ^8^ Buckingham Medical School, University of Buckingham, Buckingham, United Kingdom; ^9^ Gastroenterology and Hepatology Department, University Hospitals Coventry and Warwickshire NHS Trust, Coventry, United Kingdom; ^10^ Department of Biological Chemistry, Medical School, National and Kapodistrian University of Athens, Athens, Greece; ^11^ Endocrine Unit, 1st Department of Propaedeutic Internal Medicine, Laiko Hospital, National and Kapodistrian University of Athens, Athens, Greece; ^12^ Centre for Sport, Exercise and Life Sciences, Research Institute for Health & Wellbeing, Coventry University, Coventry, United Kingdom; ^13^ Aston Medical School, College of Health and Life Sciences, Aston University, Birmingham, United Kingdom; ^14^ College of Health, Psychology and Social Care, University of Derby, Derby, United Kingdom; ^15^ Laboratory of Dietetics and Quality of Life, Department of Food Science and Human Nutrition, School of Food and Nutritional Sciences, Agricultural University of Athens, Athens, Greece

**Keywords:** metabolic dysfunction-associated steatotic liver disease, MASLD, non-alcoholic fatty liver disease, NAFLD, metabolic dysfunction-associated fatty liver disease, MAFLD, angiopoietin-like protein 8, ANGPTL8

## Abstract

**Background:**

Steatotic liver disease related to metabolic dysfunction is the most common cause of chronic liver disease globally. The spectrum of this condition includes steatosis and steatohepatitis and was previously referred to as non-alcoholic fatty liver disease (NAFLD) but has been renamed as metabolic dysfunction-associated fatty liver disease (MAFLD) and more recently as metabolic dysfunction-associated steatotic liver disease (MASLD). Angiopoietin-like protein 8 (ANGPTL8), also known as betatrophin or lipasin, regulates triglycerides and has emerged as a potential novel biomarker for steatosis/steatohepatitis. Therefore, this systematic review aimed to identify and synthesize the evidence on the possible association of circulating ANGPTL8 concentrations with NAFLD, MAFLD or MASLD.

**Methods:**

PubMed/MEDLINE, Cochrane Library, EMBASE, and Web of Science were searched for studies published in English reporting circulating ANGPTL8 concentrations in adults with NAFLD or MAFLD or MASLD and controls. A meta-analysis was performed, reporting the standardized mean difference (SMD) of circulating ANGPTL8 concentrations between these two groups. Study quality and risk of bias were assessed using the NIH quality assessment tool and RoBANS 2, respectively.

**Results:**

Of the 104 identified publications, eight studies were eligible for this systematic review, whilst seven were also eligible for meta-analysis (543 NAFLD or MAFLD cases *vs.* 352 controls). Circulating ANGPTL8 concentrations were significantly higher in patients with NAFLD or MAFLD compared with controls (SMD: 0.62, 95%CI: 0.28-0.97; p<0.001). Considerable heterogeneity was noted among these studies, with six studies having high risk of bias in at least one RoBANS 2 domain.

**Conclusion:**

These findings present up-to-date comprehensive evidence indicating that adults with steatotic liver disease related to metabolic dysfunction exhibit higher circulating ANGPTL8 concentrations compared with controls. Given the need for novel screening/diagnostic biomarkers for steatosis/steatohepatitis, as well for additional drug targets, large and prospective studies are required to confirm this association and explore its temporal direction, particularly under the new MASLD diagnosis/term.

## 1 Introduction

Steatotic liver disease related to metabolic dysfunction is the most common cause of chronic liver disease globally, with a rising prevalence that closely follows the growing obesity and diabetes epidemic and may affect up to 30% of the general adult population (1, 2). This prevalent chronic liver disease was previously described as non-alcoholic fatty liver disease (NAFLD), but, given the well-established, strong link between NAFLD and cardio-metabolic diseases and risk factors, recent changes in nomenclature have been introduced in the relevant scientific literature (3). These include the term metabolic dysfunction-associated fatty liver disease (MAFLD), as proposed by an expert panel in 2020, and more recently the term metabolic dysfunction-associated steatotic liver disease (MASLD) as proposed by a multi-society Delphi statement in 2023 (4, 5), thus highlighting the key role of cardio-metabolic diseases/dysfunction in the pathophysiology of this chronic liver disease. This new nomenclature also introduced the term metabolic and alcohol related/associated liver disease (MetALD) for those patients with MASLD and a weekly alcohol intake greater than 140-350 g and 210-420 g for females and males, respectively (4, 5).

**Figure 1:**
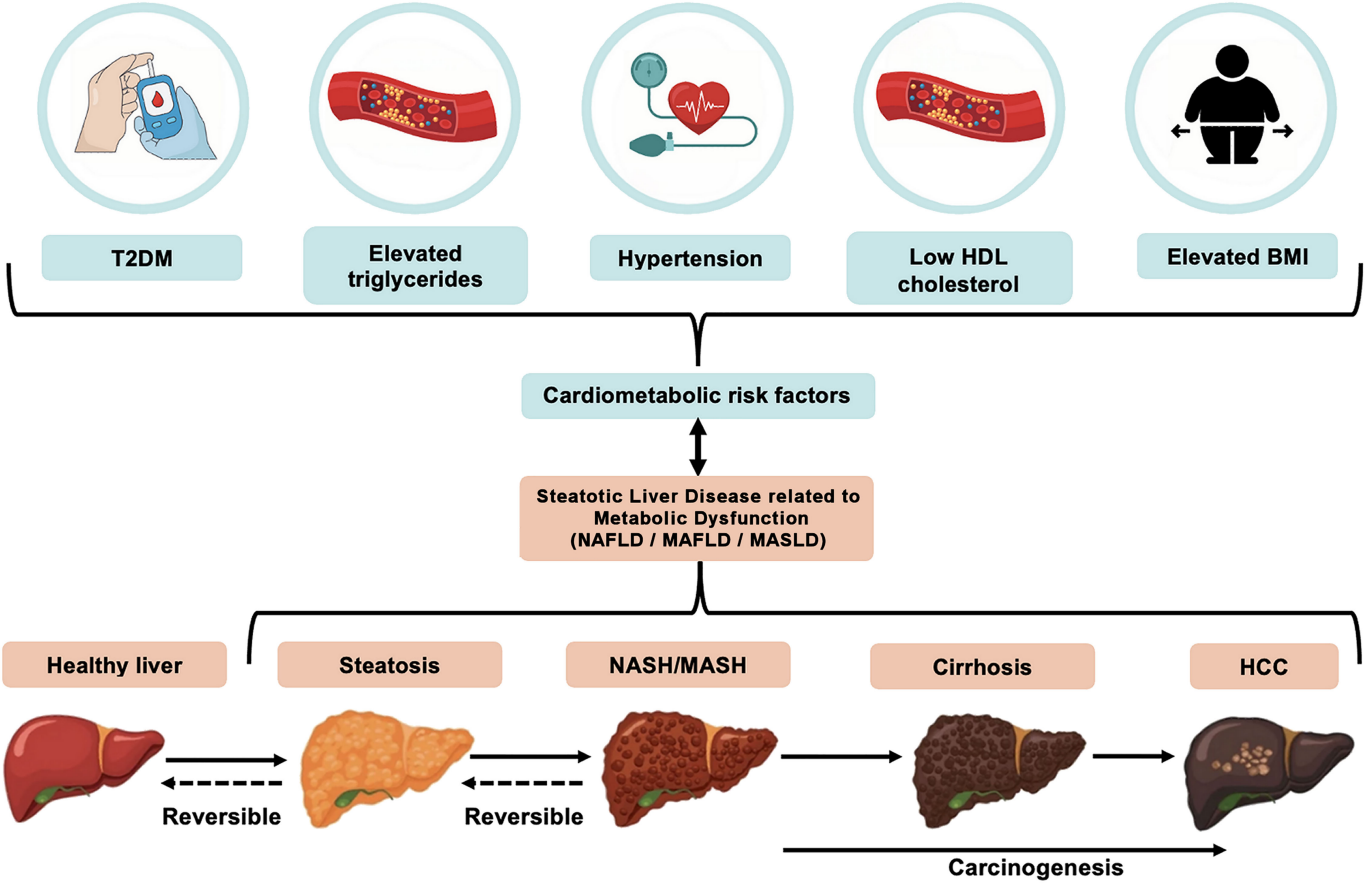
The relationship between key cardio-metabolic diseases/risk factors and disease development and progression of non-alcoholic fatty liver disease (NAFLD), more recently referred to as metabolic dysfunction-associated steatotic liver disease (MASLD). MASLD is diagnosed on the presence of hepatic steatosis combined with at least one of the five following cardio-metabolic diseases/risk factors: elevated body mass index (BMI), increased fasting glucose levels or type 2 diabetes (T2DM), hypertension, increased triglycerides, or low HDL-cholesterol levels. The MASLD spectrum ranges from simple hepatic steatosis (>5% fat accumulation within hepatocytes), to steatohepatitis [metabolic-associated steatohepatitis (MASH), previously referred to as non-alcoholic steatohepatitis (NASH)], where inflammation and ballooning of hepatocytes and/or hepatic fibrosis are noted. This may then progress to cirrhosis and even hepatocellular carcinoma (HCC) where prognosis is typically very poor.

MASLD, which now represents the preferred adopted nomenclature/term, encompasses a disease spectrum ranging from simple steatosis to metabolic-associated steatohepatitis (MASH; previously referred to as non-alcoholic steatohepatitis, NASH), which may further progress to cirrhosis and hepatocellular carcinoma (HCC) (**Figure 1**). The exact natural history of this disease remains to be fully understood, with evidence revealing a complex trajectory where the disease can potentially progress to more severe forms (e.g., MASH and cirrhosis) or remain at a benign state (6, 7). The majority of patients with hepatic steatosis may, at least initially, lack specific symptoms and/or biochemical abnormalities; thus, the onset of this disease is generally clinically silent, and its diagnosis may be delayed until the development of complications. Although liver biopsy remains the gold standard for diagnosis and staging, a combination of non-invasive biomarkers, imaging, and scoring systems are currently used for the screening and diagnosis of steatosis/steatohepatitis, which are collectively termed by the American Association for the Study of Liver Disease (AASLD) as noninvasive liver disease assessment(s) (3, 8–12). Indeed, liver biopsy is limited by its invasive nature, sampling variability, and impracticality for population screening, which has driven the broad search for non-invasive serum biomarkers for screening and early disease diagnosis and monitoring (13).

Angiopoietin-like proteins (ANGPTLs) are a family of eight circulating glycoproteins (ANGPTL1 to ANGPTL8) with a common structure and also specific features that differentiate their role in tissue expression and regulation (14). ANGPTLs play a significant role in lipid metabolism, insulin resistance, and hormone regulation, and may be an important link to the metabolic syndrome disease process (15, 16). Specifically, ANGPTL8, also known as betatrophin or lipasin, is considered as a key emerging player in lipid metabolism, regulating triglyceride levels by affecting the activity of lipoprotein lipase (16). Notably, epidemiological studies have demonstrated an association between ANGPTL8 and metabolic diseases (17–19), including hepatic steatosis; thus, posing it as a potential biomarker for diagnosis and monitoring. However, the potential association between circulating ANGPTL8 with steatosis/steatohepatitis has yet to be established due to inconsistent study results and the recent changes to the diagnostic definition of MAFLD and MASLD. Therefore, the present systematic review aimed to synthesise the existing relevant studies and meta-analyze the available data on the differences in circulating levels of ANGPTL8 between patients with NAFLD or MAFLD or MASLD and controls in order to investigate its potential utility as a serum biomarker for the diagnosis and monitoring of steatosis/steatohepatitis.

## 2 Methods

This systematic review was conducted in accordance with the Preferred Reporting Items for Systematic Reviews and Meta-Analyses (PRISMA) guidelines (**Supplementary Table 1**) and was registered in the PROSPERO International Prospective Register of Systematic Reviews (CRD42024584720).

### 2.1 Search strategy and data sources

A comprehensive literature search was conducted based on predefined search strategies in Medline (via PubMed), Cochrane Library, Embase, and Web of Science (core collection). We also searched clinicaltrials.gov and the reference lists of relevant publications to ensure literature saturation. The final searches were completed in June 2024. The search strategy used for Medline (via PubMed) (**Table 1**) was adapted to the syntax and appropriate subject headings of the other databases (**Supplementary Table 2**).

**Table 1:** Search strategy for Medline (via PubMed).

(“Metabolic Dysfunction-associated Steatotic Liver Disease” OR MASLD OR “non-alcoholic fatty liver disease” OR (NAFLD OR “Non-alcoholic fatty liver disease”[MeSH Terms] OR (“non alcoholic” AND fatty AND liver AND disease) OR “non-alcoholic fatty liver disease” OR NAFLD) OR “Metabolic Dysfunction-associated Fatty Liver Disease” OR MAFLD) AND (“Angiopoietin-like 8” OR ((“angiopoietin like protein 8”[MeSH Terms] OR “angiopoietin like protein 8” OR Angiopoietin-like) AND 8) OR ANGPTL8 OR TD26 OR RIFL OR Lipasin OR (“angiopoietin like protein 8”[MeSH Terms] OR “angiopoietin like protein 8” OR betatrophin))

#### 2.1.1 Clarification regarding the applied nomenclature/terms

The new nomenclature/terms that have been introduced for NAFLD reflect the strong relationship of this disease with cardio-metabolic risk factors/diseases (4, 5). In contrast to the diagnosis of NAFLD, which is defined by the absence of other liver conditions (e.g., viral hepatitis, autoimmune or alcoholic liver disease), MASLD is diagnosed on the presence of hepatic steatosis (i.e., >5% fat accumulation within hepatocytes) and the presence of at least one of five cardio-metabolic risk factors [i.e., elevated body mass index (BMI), raised fasting glucose levels or type 2 diabetes mellitus (T2DM), hypertension, raised triglycerides, or low high-density lipoprotein (HDL)-cholesterol levels], as summarized in **Figure 1** (5). Similar criteria have been proposed for MAFLD (4). MASLD also eliminates from the relevant nomenclature the term “fatty” which was considered stigmatizing (5, 20). Notably, the proposal of these new terms/nomenclature has also raised certain concerns regarding the interchangeability of the terms NAFLD, MAFLD and MASLD, and accordingly the inter-application of the corresponding evidence (21). Although the same pathophysiological mechanisms are likely to underpin NAFLD, MAFLD and MASLD (3), research studies which have specifically explored MAFLD and MASLD cohorts are still limited due to the recent introduction/definition of these terms. Therefore, for the purposes of the present systematic review, the applied search strategies searched for all three existing terms, namely NAFLD, MAFLD and MASLD, and in the following sections where primary research studies are cited the corresponding NAFLD, or MAFLD, or MASLD term is used in the present paper as stated/defined in the corresponding published paper.

### 2.2 Eligibility criteria

Eligible studies included those conducted in adults (≥18 years of age) and reporting circulating levels of ANGPTL8 in a relevant disease (i.e., NAFLD or MAFLD or MASLD) and control group. An English language restriction was applied. No restrictions were imposed regarding the year of publication or the type of healthcare setting. All analytical observational study designs were considered eligible, while descriptive observational studies (i.e., case reports, case series, case studies), expert opinion manuscripts, commentaries, animal studies, and review articles were excluded.

### 2.3 Study selection and data extraction

The study selection and data extraction were completed independently by FA and LL. Both the initial screening (title and abstract) and screening of full text articles were performed using the Rayyan software (22). A standardized data extraction form was developed to capture relevant information. This included the country of origin, study design, patient demographics and the relevant outcomes (i.e., circulating levels of ANGPTL8).

### 2.4 Risk of bias and assessment of study quality

The risk of bias and quality assessments were completed independently by two reviewers (FA and LL). For the risk of bias and quality assessment, the Revised Risk of Bias Assessment Tool for Nonrandomized Studies (RoBANS 2) was used, which covers seven domains (i.e., comparability of the target group; target group selection; confounders; measurement of exposure; blinding of assessors; incomplete outcome data; and selective outcome reporting) (23). Additionally, quality was also assessed based on the National Institute of Health (NIH) Study Quality Assessment Tool (24).

### 2.5 Statistical analysis

Where possible, effect sizes were pooled in a random effects meta-analysis. Hedges’ g was used as the measure of effect size due to its correction for small sample bias, providing a more consistent estimate across studies compared to other effect size measures such as Cohen’s d (25). The pooled Hedges’ g and its 95% confidence intervals (CIs) were calculated using the Knapp-Hartung adjustment method. This approach accounts for the uncertainty in between-study variance to provide more conservative CIs particularly in the presence of high heterogeneity or when few studies are included in the analysis (26, 27). The meta-analysis was performed in R Studio (2024.04.2 + 764 “Chocolate Cosmos” for Windows, R version: R-4.4.1) using the “meta” package version 7.0-0 (28).

Heterogeneity among studies was assessed using the *I²* statistic. The *I^2^* statistic describes the percentage of variability between point estimates which is attributable to heterogeneity, rather than sampling error. Accordingly, heterogeneity was interpreted as: 0-40% may not be important, 30-60% may indicate moderate heterogeneity, 50-90% may represent substantial heterogeneity, and 75-90% as considerable heterogeneity (29). Subgroup analyses by reported severity of steatosis/steatohepatitis and diagnostic method were performed to explore potential sources of heterogeneity and their impact on the pooled effect size. We also regressed the difference in BMI between the disease and control groups within each study to further examine potential moderators influencing the pooled effect. Sensitivity analyses were performed to explore sources of heterogeneity and identify outliers, which included: removal of studies that were judged as “poor” quality, visual inspection of a Baujat plot, and an influence analysis diagnostics proposed by Viechtbauer and Cheung (27) and implemented using the dmetar package (version 0.1.0) (30). Publication bias was evaluated using Egger’s test and visual inspection of the funnel plot (31). Using the “metaviz” package version 0.3.1 (32), a sunset power-enhanced funnel plot was used to visually represent the statistical power of the included studies in relation to the pooled effect size. A p-curve analysis was conducted using the dmetar package to assess the evidential value of the included studies to determine if the significant findings are likely due to true effects rather than publication bias or data manipulation bias (33–35).

## 3 Results

### 3.1 Study selection

**Figure 2:**
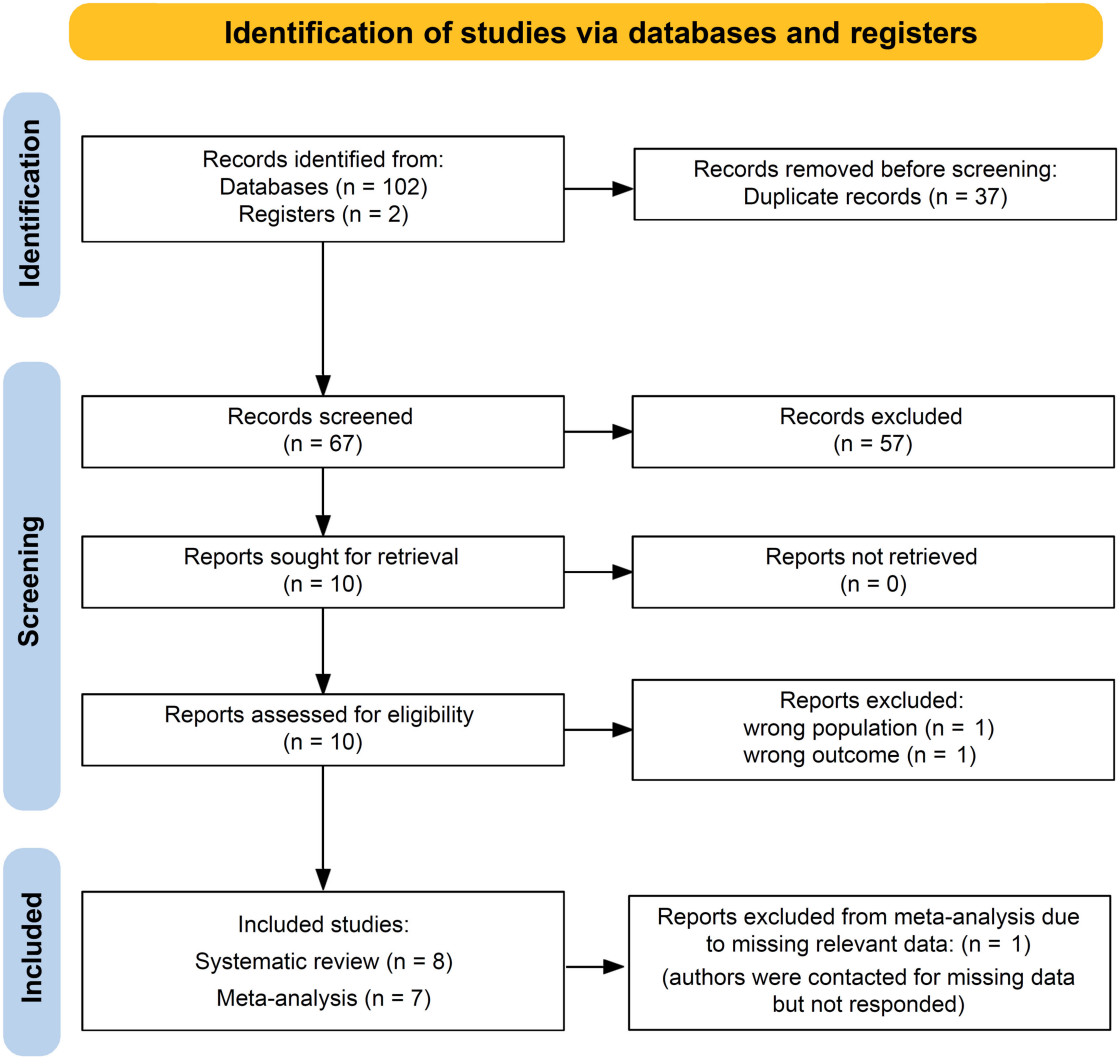
PRISMA flow diagram depicting the study selection process.

A total of 104 studies were identified across the databases/sources searched, and 37 duplicates were removed. The remaining 67 articles underwent title and abstract screening, and 57 studies were considered irrelevant, leaving ten articles for full-text review. During full-text review, two studies were excluded with reasons (**Figure 2**), resulting in eight studies eligible for inclusion (36–43).

All included studies were observational in design, comprising cross-sectional, cohort, and case-control studies. Of these, four studies originated from China (36, 39, 41, 42), two from Turkey (38, 43) and one each from Germany (37) and South Korea (40) (**Table 2**, **Supplementary Figure 1** also presents a map of the world with the location/country of origin for the studies included in this systematic review). All studies applied the NAFLD diagnosis [except the 2021 study by Sönmez et al. (38), all these studies were published before the introduction of the MAFLD and MASLD nomenclature in 2020 and 2023, respectively], except the 2024 study by Gan et al. (42) which applied the MAFLD diagnosis (**Table 2**). None of the eligible studies applied the MASLD diagnosis. NAFLD/MAFLD status was diagnosed by liver biopsy in three studies (37, 38, 43), whilst in the remaining five studies the diagnosis was based on assessment by ultrasonography (36, 42), ultrasonography or computed tomography (CT) (40), magnetic resonance imaging (MRI that included localizer images and the T1 volumetric interpolated breath-hold examination Dixon sequence to calculate the hepatocellular lipid content; a hepatocellular lipid content >5.5% was considered diagnostic for NAFLD; mild NAFLD: 5.5-10.0% hepatocellular lipid content and moderate-to-severe NAFLD ≥10.0% hepatocellular lipid content) (39) or a combination of those with biochemical tests (41). All but one of the eligible studies reported circulating levels of ANGPTL8 using mean and standard deviation (SD), while the study by Cengiz et al. (43) reported these data using median and standard error, which meant that accurately transforming these data was not feasible. Attempts to contact the authors of the latter study were unsuccessful, so this study was excluded from the meta-analysis.

**Table 2:** Key characteristics of the eight eligible studies included in the present systematic review.

Author (Year)	Study characteristics	NAFLD/MAFLD group	Control group	Key findings
Hu et al. (2017) (36)	Country: China Design: Cross-sectional ELISA: Beijing Cheng Lin Biological Technology Inc. Diagnosis: NAFLD Diagnostic Method: Ultrasonography (assessment of liver fat content by quantitative ultrasonography with a cut-off value of 9.15% for NAFLD diagnosis)	N: 165 Sex: 53% female Age: 59.7 ± 0.4 years BMI: 26.5 ± 0.2 kg/m^2^	N: 84 Sex: 49% female Age: 60.4 ± 0.7 years BMI: 23.6 ± 0.3 kg/m^2^	Serum ANGPTL8 levels are significantly higher (*p* = 0.035) in NAFLD patients (279.1 ± 118.6 pg/mL) compared to controls (254.5 ± 109.2 pg/mL).
von Loeffelholz et al. (2017) (37)	Country: Germany Design: Cross-sectional ELISA: Wuhan EIAab Science Co. Ltd. Diagnosis: NAFLD Diagnostic Method: Liver biopsy	N: 24 Sex: 54% female Age: 60.0 ± 14.7 years BMI: 27.0 ± 6.9 kg/m^2^	N: 16 Sex: 69% female Age: 54.0 ± 16.0 years BMI: 23.8 ± 3.6 kg/m^2^	Serum ANGPTL8 levels were not significantly different (*p* = 0.80) in the NAFLD (1213.9 ± 996.9 pg/mL) compared to the control (1016.5 ± 764.4 pg/mL) group.
Sönmez et al. (2021) (38)	Country: Turkey Design: Cross-sectional ELISA: Wuhan EIAab Science Co. Ltd. Diagnosis: NAFLD Diagnostic Method: Liver biopsy	N: 50 Sex: 100% male Age: 32.3 ± 5.5 years BMI: 28.4 ± 2.1 kg/m^2^ [non-NASH (simple steatosis and borderline NASH with NAFLD activity score <5): N=18; and NASH (NAFLD activity score ≥5): N=32]	N: 30 Sex: 100% male Age: 28.5 ± 3.9 years BMI: 24.9 ± 1.4 kg/m^2^	Higher circulating ANGPTL8 levels in the early stages of NAFLD, which tend to decrease as the disease progresses. Serum ANGPTL8 levels were 271.0 ± 449.3 pg/mL in NAFLD patients (non-NASH and NASH group combined) and 93.22 ± 55.35 pg/mL in controls. Serum ANGPTL8 levels were statistically different (*p* = 0.05) when the controls and the two NAFLD groups (non-NASH and NASH) were compared, with significant differences lying between the control and non-NASH group (*p* = 0.02).
Hong et al. (2018) (39)	Country: China Design: Cross-sectional ELISA: Wuhan EIAab Science Co. Ltd. Diagnosis: NAFLD Diagnostic Method: MRI	N: 36 Sex: 58% female Age: 54.1 ± 6.2 years BMI: 25.1 ± 2.25 kg/m^2^ [Mild NAFLD (≥5.5 to 10.0% hepatocellular lipid content on the MRI): N=18; and Moderate-to-severe NAFLD (≥10.0% hepatocellular lipid content on the MRI): N=18]	N: 12 Sex: 50% female Age: 52.2 ± 4.8 years BMI: 23.6 ± 1.7 kg/m^2^	Higher serum ANGPTL8 levels were present in the moderate-to-severe NAFLD group compared to the non-NAFLD and mild NAFLD groups (1,129 ± 351 vs 742 ± 252 and 765 ± 301 pg/mL, respectively, *p* = 0.001). ANGPTL8 levels were comparable between the non-NAFLD and mild NAFLD groups.
Lee et al. (2016) (40)	Country: South Korea Design: Cross-sectional ELISA: Phoenix Pharmaceuticals Inc. Diagnosis: NAFLD Diagnostic Method: Ultrasonography/CT	N: 96 Sex: 41% female Age: 52.4 ± 12.8 years BMI: 26.7 ± 4.1 kg/m^2^	N: 38 Sex: 53% female Age: 56.3 ± 10.4 years BMI: 23.4 ± 3.3 kg/m^2^	Serum ANGPTL8 levels were significantly higher (*p <*0.001) in NAFLD patients (1301.0 ± 617.0 pg/mL) compared to controls (900.0 ± 574.0 pg/mL), even after stratification by obesity or diabetic status.
Zhu et al.(2016) (41)	Country: China Design: Case-matched cross-sectional^††^ ELISA: Wuhan EIAab Science Co. Ltd. Diagnosis: NAFLD Diagnostic Method: Ultrasonography plus biochemical tests	N: 92 Sex: 35% female Age: 55.1 ± 12.0 years BMI: 24.7 ± 3.9 kg/m^2^	N: 92 Sex: 35% female Age: 53.1 ± 10.1 years BMI: 24.4 ± 3.0 kg/m^2^	Serum ANGPTL8 levels were significantly higher (*p <*0.001) in NAFLD patients (1095.0 ± 541.3 pg/mL) compared to controls (730.0 ± 431.1 pg/mL).
Gan et al. (2024) (42)	Country: China Design: Cross-sectional^††^ ELISA: Wuhan Huamei Biological Engineering Inc. Diagnosis: MAFLD Diagnostic Method: Ultrasonography	N: 80 Sex: 41% female Age: 54.7 ± 12.2 years BMI: 25.0 ± 3.6 kg/m^2^	N: 80 Sex: 46% female Age: 55.4 ± 12.8 years BMI: 22.3 ± 3.4 kg/m^2^	Serum ANGPTL8 levels were significantly higher (*p <*0.001) in MAFLD patients (45.5 ± 18.6 pg/mL) compared to controls (24.9 ± 13.4 pg/mL).
Cengiz et al. (2016) (43)	Country: Turkey Design: Case-matched cross-sectional ELISA: Aviscera Bioscience Inc. Diagnosis: NAFLD Diagnostic Method: Liver biopsy	N: 69 Sex: 54% female Age: 48.4 ± 11.2 years BMI: 29.8 ± 4.3 kg/m^2^	N: 69 Sex: 55% female Age: 49.1 ± 10.8 years BMI: 27.3 ± 4.3 kg/m^2^	Serum ANGPTL8 levels were significantly lower (*p =* 0.001) in NAFLD patients (1940.0 ± 90.0^†^ pg/mL) compared to controls (2340.0 ± 60.0^†^ pg/mL). The mild fibrosis group (fibrosis score less than 2) had higher serum ANGPTL8 levels than the significant fibrosis group (fibrosis score of 2 or greater), and so ANGPTL8 levels tend to decrease when the disease progresses.

BMI, body mass index; ELISA, enzyme-linked immunosorbent assay; CT, computed tomography; MAFLD, metabolic dysfunction-associated fatty liver disease; MRI, magnetic resonance imaging; NAFLD, non-alcoholic fatty liver disease. All data are presented as mean ± standard deviation (with the exception for the Cengiz et al. (2016) (43) study where ^†^ indicates that these data were reported as median and standard error) and rounded to one decimal place; ^††^ indicates that these two studies reported a case-control study design in the title and/or methods of the corresponding papers, although we discerned that these were cross-sectional.

The eight eligible studies included in the present systematic review involved a total of 612 NAFLD/MAFLD cases and 421 controls. The proportion of female participants in the NAFLD/MAFLD groups ranged between 0% (38) and 58% (39), whilst the average age of participants ranged from 32.3 (38) to 60.0 (37) years, and the BMI from 25.1 (39) to 29.8 (43) kg/m². In comparison, the age and BMI range for controls was slightly lower compared to the cases ranging from 28.5 (38) to 60.4 (36) years, and 23.4 (40) to 27.3 (43) kg/m², respectively. However, a similar percentage of female participants [0% (38) to 55% (43)] was reported amongst controls. Further details on study design, population characteristics, disease, and ANGPTL8 assessment for each of the included eight studies are presented in **Table 2**.

### 3.2 Risk of bias and study quality assessment

**Figure 3:**
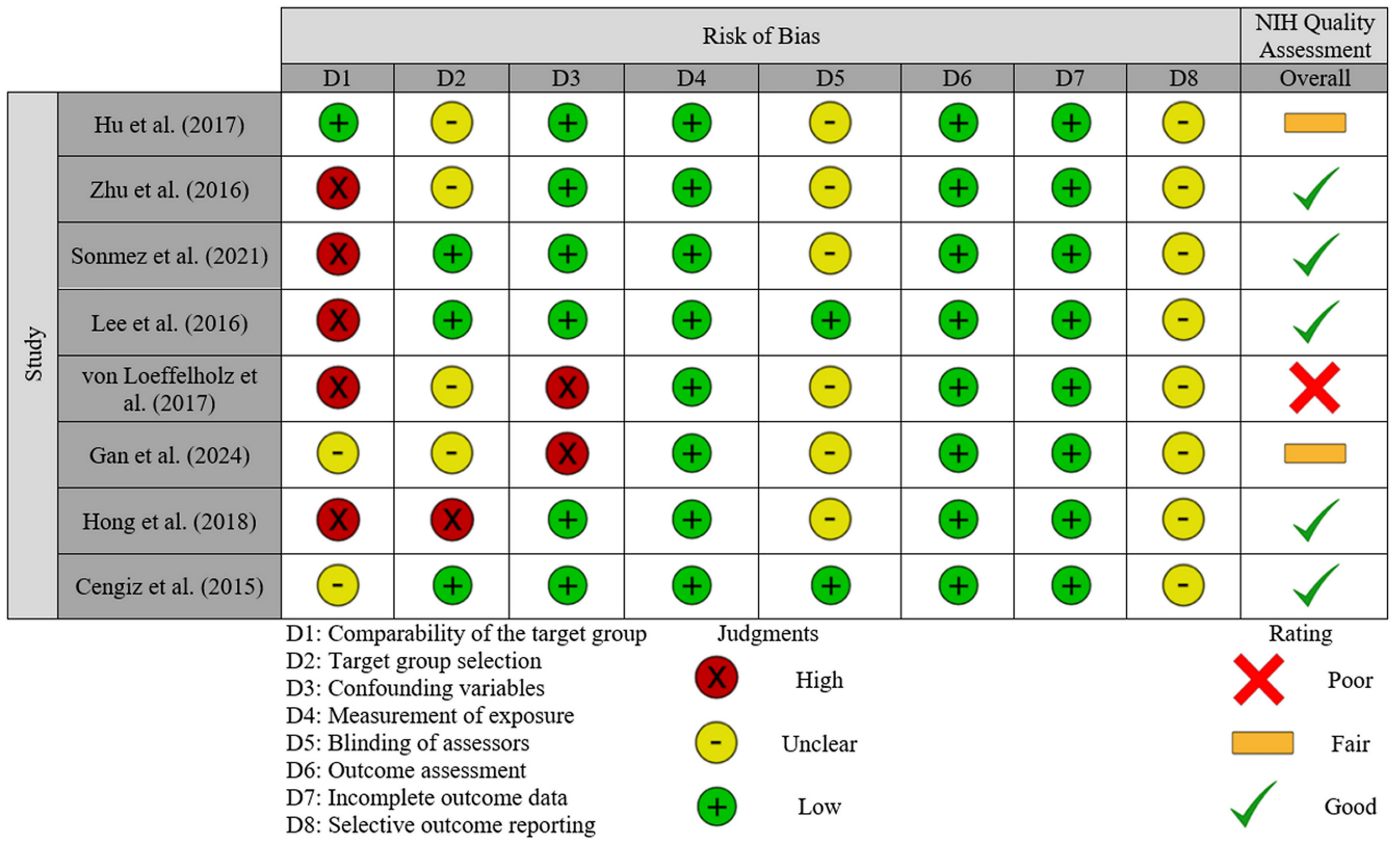
Risk of bias and quality assessment summary: review of judgements on each item/domain (D1 to D8) from the Risk of Bias Assessment Tool for Nonrandomized Studies (RoBANS 2) presented as high, unclear or low risk across all included studies. An overall quality assessment based on the NIH Quality assessment tool is also included for each study, presented as good, fair or poor.

The risk of bias judgments and assessment of study quality are presented in **Figure 3**. For the three domains relating to selection bias, five studies (62.5%) were judged to have a high risk of bias when comparing the target group (37–41); this was largely due to substantial differences between NAFLD and control participants. Two studies (42, 43) were judged as having an unclear risk for this domain, whilst the remaining study had a low risk for this domain. For target group selection, the study by Hong et al. (39) was deemed to have a high risk of bias since all recruited individuals, including those in the control group, were recruited as having high-risk of NAFLD based on the presence of obesity, T2DM, metabolic syndrome, or abnormal plasma aminotransferases. Four studies (36, 37, 41, 42) were judged to have an unclear risk as participant selection methods were not sufficiently reported. The remaining three studies were judged as having a low risk. All studies included confounding variables, but only two (37, 42) were judged as high risk, due to concerns about how these were accounted for in analyses [e.g., only adjusted for age (37) or did not adjust for confounders (42)]; the remaining studies were all deemed low risk since their statistical models exploring the association with ANGPTL-8 accounted for a number of potential confounders. Although liver biopsy is the gold standard for diagnosis of steatosis/steatohepatitis, since all included studies applied established methods for diagnosing NAFLD/MAFLD which are used in routine clinical practice, performance bias (i.e., measurement of exposure) was judged to be low risk in all these studies. For bias related to blinding of assessors, six studies (36–39, 41, 42) did not report whether assessors were blinded so were judged as having an unclear risk; the remaining two studies had a low risk. All studies used commercially available ELISA kits to measure ANGPTL8 (**Table 2**), so were deemed as having a low risk for this domain. Similarly, attrition bias was judged as low in all studies since there was very little missing data reported. Finally, reporting bias, due to selective outcome reporting, was judged as unclear for all studies since identifying any registry records for any of the included studies was not possible.

When study quality was assessed (NIH Quality assessment in **Figure 3**), five studies were rated as good (38–41, 43), two were rated as fair (36, 42), and one was judged as poor (37). Common reasons for downgrading quality were lack of sample size justification, variations in how exposure severity was managed, failures to report blinding of assessors, and the management of confounding variables.

### 3.3 Circulating ANGPTL8 levels and NAFLD/MAFLD

**Figure 4:**
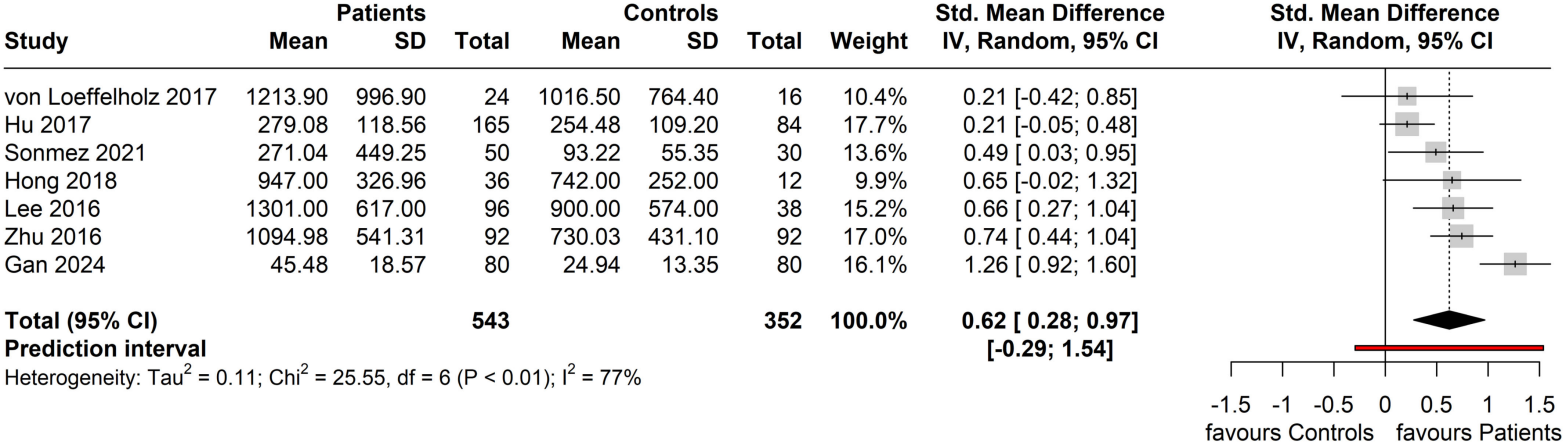
Forest plot of circulating ANGPTL8 levels amongst patients with NAFLD or MAFLD and controls [Random-Effects model; Standardized (Std.) Mean Difference is represented as Hedge’s g].

For the seven included studies that were eligible for meta-analysis, the performed random-effects meta-analysis produced a Hedge’s g summary effect of 0.62 (95% CI: 0.28, 0.97), showing that patients with NAFLD/MAFLD had statistically higher circulating ANGPTL8 levels compared to the controls (p = 0.004) (**Figure 4**). Whilst a statistical effect was reported, considerable between-study heterogeneity was observed (*I^2^* = 77%; Q = 25.55 (df = 6), p < 0.001).

### 3.4 Subgroup analysis

**Figure 5:**
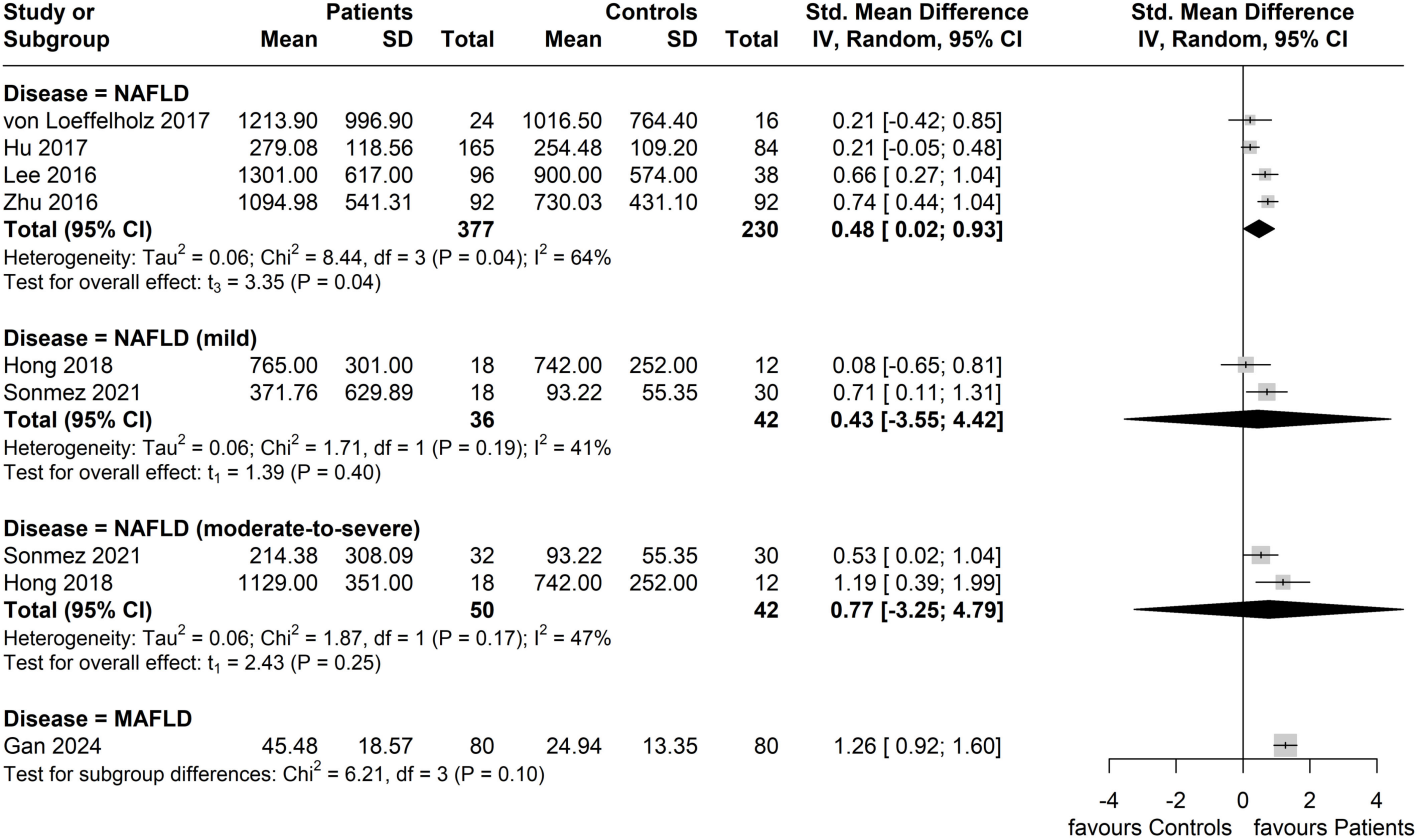
Forest plot of circulating ANGPTL8 levels amongst patients with NAFLD or MAFLD and controls by disease severity as/where reported in the included eligible studies [Random-Effects model; Standardized (Std.) Mean Difference, SMD].

Two of the six studies that applied the NAFLD diagnosis further specified the severity of NAFLD; the study by Sönmez et al. (38) categorized cases as non-NASH (simple steatosis and borderline NASH, corresponding to mild NAFLD) and NASH (corresponding to moderate-to-severe NAFLD) based on a NAFLD activity score (NAS) of <5 and ≥5, respectively, whilst the study by Hong et al. (39) categorized mild and moderate-to-severe NAFLD based on the presence of, respectively, 5.5-10.0% and ≥10.0% hepatocellular lipid content according to the performed MRI. The results of the performed subgroup analysis by disease severity (where reported in included studies) are shown in **Figure 5**. In the mild NAFLD disease subgroup, no significant difference in circulating ANGPTL8 levels was noted compared to the controls (Standardized Mean Difference, SMD = 0.43, 95% CI: -3.55, 4.42, *p* = 0.40; *I^2^* = 41%). Similarly, circulating ANGPTL8 levels were not significantly higher amongst patients in the NAFLD moderate-to-severe subgroup compared to the controls (SMD: 0.77, 95% CI: -3.25, 4.79, *p* = 0.25; *I^2^* = 47%). However, in the studies that did not specify NAFLD severity, significantly higher circulating ANGPTL8 levels were observed in the patients with NAFLD compared to controls (SMD: 0.48, 95% CI: 0.02, 0.93. *p* = 0.04); although, there was substantial heterogeneity (*I^2^* = 64%). Furthermore, when regressed, BMI was not a statistically significant moderator (p = 0.56).

**Figure 6:**
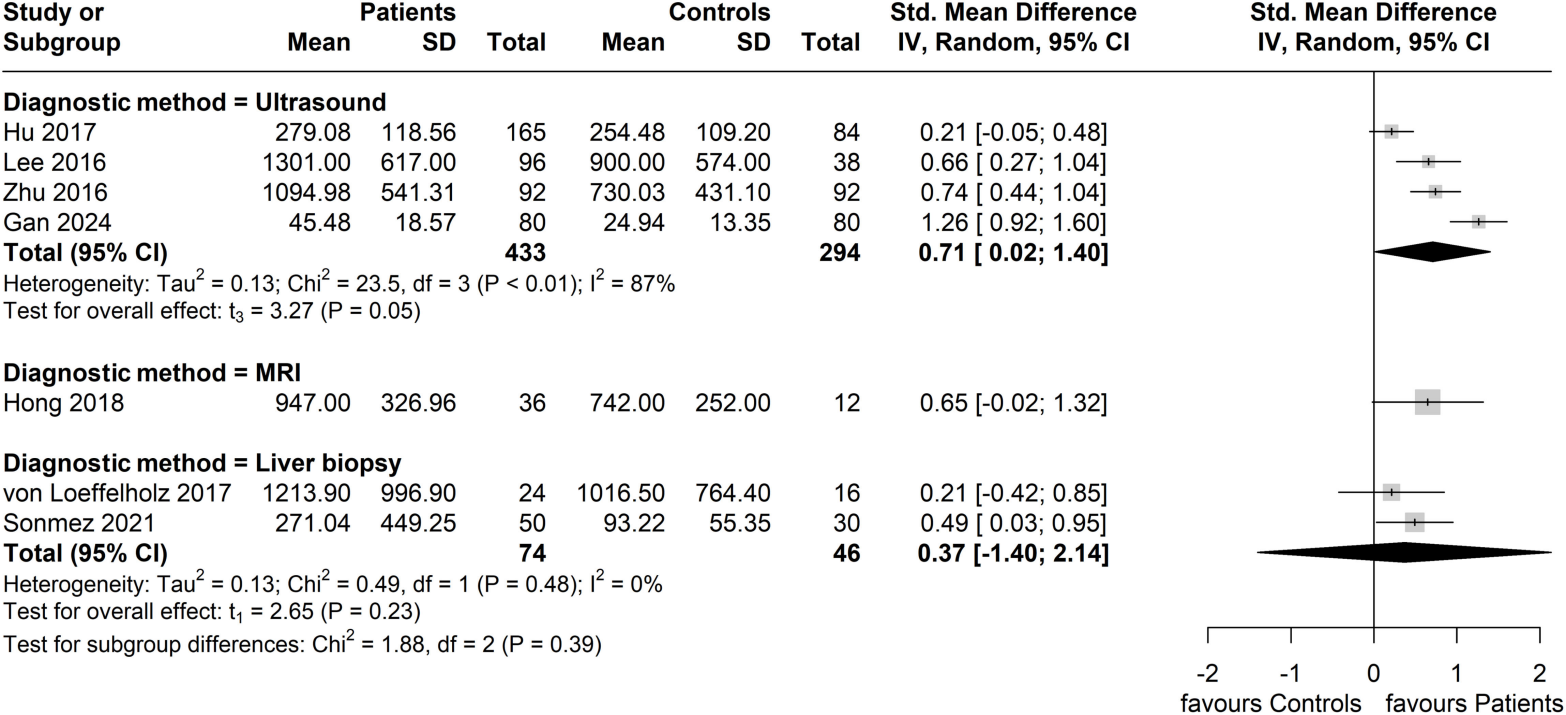
Forest plot of circulating ANGPTL8 levels amongst patients with NAFLD or MAFLD and controls by applied method of diagnosis in the included studies [Random-Effects model; Standardized (Std.) Mean Difference, SMD]. For the purposes of this subgroup analysis, we included the study by Lee et al. (40) in the group of studies applying ultrasonography, although 13% of the study participants (17 of 134) were screened for NAFLD by computed tomography.

In subgroup analysis based on the method of NAFLD/MAFLD diagnosis, the greatest effect size was seen in the subgroup with an ultrasound-based diagnosis (four studies, n = 433 patients; n = 294 controls), with a pooled SMD of 0.71 (95% CI: 0.02, 1.40). However, this subgroup also exhibited the highest heterogeneity (*I^2^* = 87%). No significant difference between patients and controls (two studies, n = 74 patients; n = 46 controls) was observed in the liver biopsy diagnosed subgroup (SMD = 0.37; 95% CI: -1.40, 2.14; *I^2^* = 0%), whilst only one study was included in the MRI-based diagnosis subgroup (**Figure 6**).

**Figure 7:**
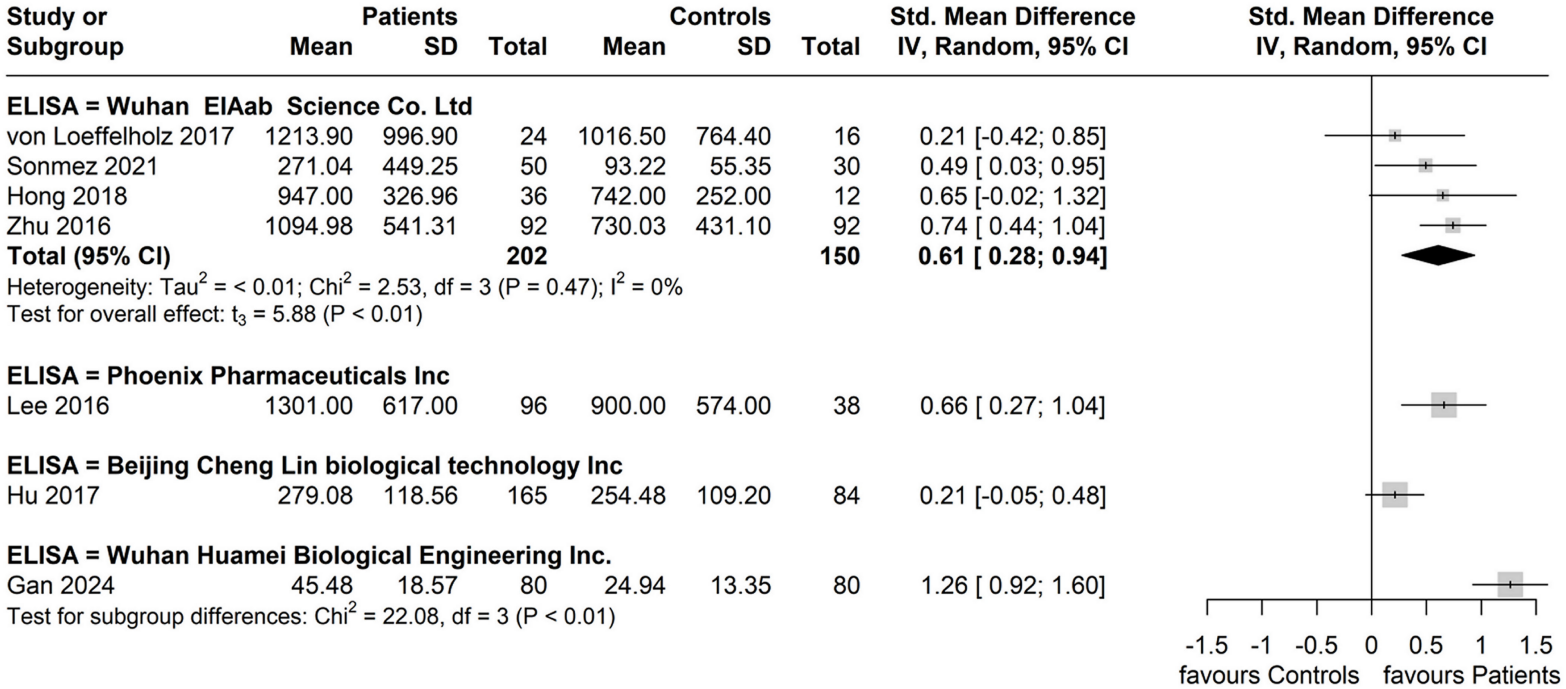
Forest plot of circulating ANGPTL8 levels patients with NAFLD or MAFLD and controls by reported ELISA kit used for ANGPTL8 measurement [Random-Effects model; Standardized (Std.) Mean Difference, SMD].

When considering ANGPTL8 measurement methods, the subgroup reporting having used the Wuhan EIAab Eliza kit had higher circulating ANGPTL8 levels amongst patients with NAFLD compared to controls with diminished heterogeneity between these four studies (SMD: 0.61, 95% CI: 0.28, 0.94. *p* < 0.01; *I^2^* = 0%) (37–39, 41). For the remaining reported ELISA kits, each subgroup did not have more than one study for calculation of a pooled effect (**Figure 7**).

### 3.5 Sensitivity analysis

Gan et al. (42) was identified as an outlier; once this study was removed, the summary effect was reduced, but remained significant (SMD = 0.50, 95% CI: 0.24, 0.75, p < 0.01). Removing studies which did not define the patient group as NAFLD, but as MAFLD, yielded the same results, as Gan et al. (42) was the only such study. Removal of this study also resulted in substantial reduction in the overall heterogeneity (*I^2^* reduced to 43% from 77%). Removing the only study (37) identified as ‘Poor’ according to the NIH quality assessment, resulted in an increase in the overall SMD to 0.67 (95% CI: 0.29, 1.05, p = 0.006), although considerable heterogeneity (*I^2^* = 79%) remained. Results of the sensitivity and outlier analyses are presented in **Supplementary Figures 2–4**.

### 3.6 Publication bias

**Figure 8:**
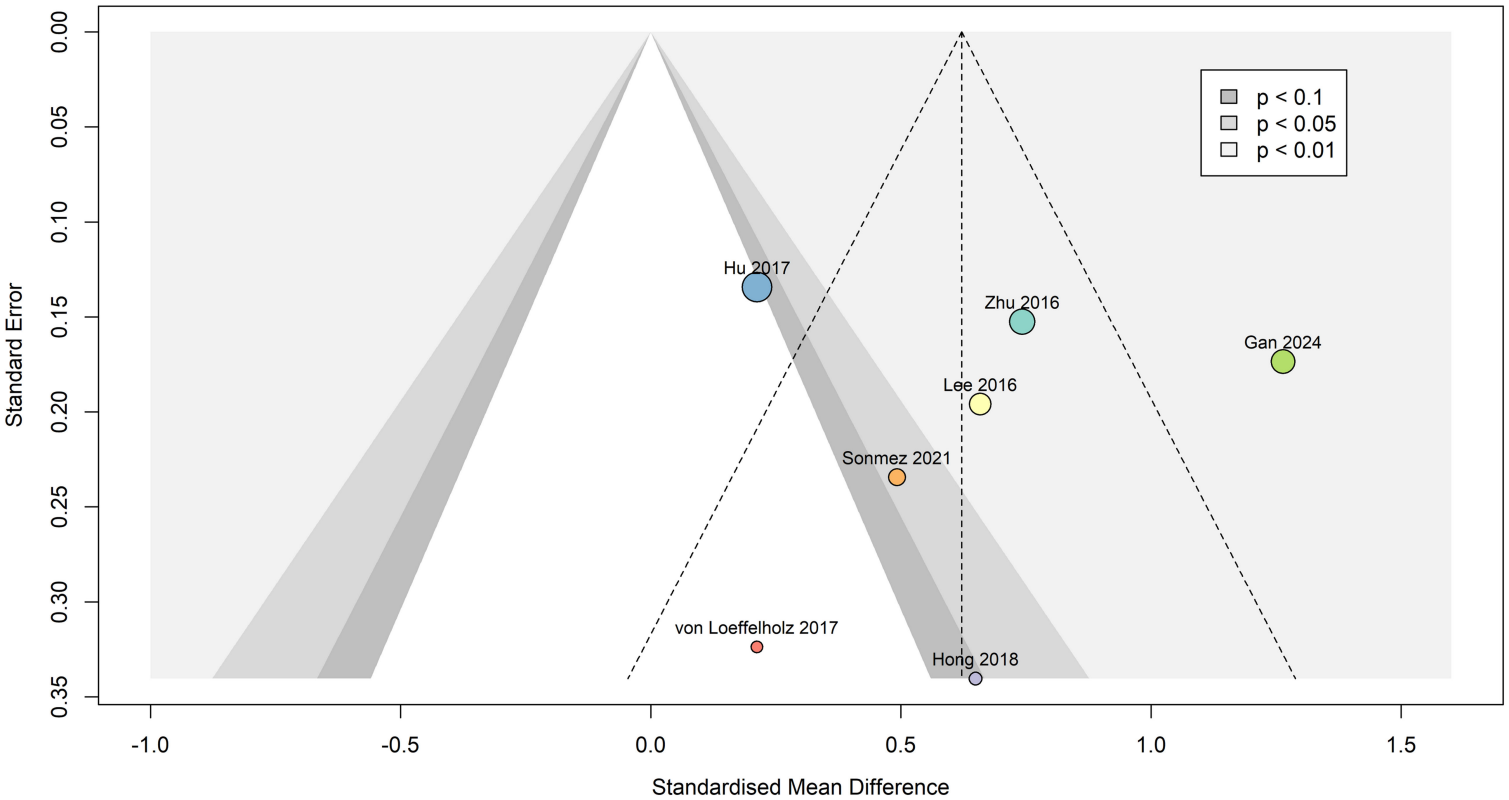
Contour-enhanced funnel plot indicating that publication bias was not present. Dots are scaled based on study sample size. Standardized mean difference is represented as Hedges’ g, and the summary effect was calculated using the random effects model.

Egger’s publication bias test indicated that funnel plot asymmetry was not likely present (β_0_ = 0.188 [-5.4, 5.78], t = 0.066, p = 0.95), as seen in **Figure 8**. Due to absence of asymmetry in the funnel plot, the Duval and Tweedie’s trim and fill test was not performed.

The power-enhanced sunset plot (**Supplementary Figure 5**) showed that four (36, 40–42) out of the seven meta-analyzed studies had sufficient power (> 80%) to detect the underlying true effect of interest (i.e., the meta-analytic summary effect). However, one (36) out of these four studies’ effects was not significantly different to zero. The median power of all studies included was 88.8%.

The results of the p-curve analysis indicated that selective reporting or data manipulation was unlikely, as four of the five significant effects were highly significant (p < 0.025). The p-curve shows a right-skew (**Supplementary Figure 6**), which suggests a true effect is likely. Overall, this indicates evidential value and the presence of a genuine non-zero effect. Although we cannot entirely rule out publication bias, the significant right-skew and nonsignificant flatness tests (p < 0.05 and p > 0.05, respectively) and high power (99%) support that the pooled effect in our meta-analysis was not likely to be due to selective reporting, data manipulation or a spurious association. Rather, all significant results were not false positives.

## 4 Discussion

Of the eight studies included in the present systematic review, seven studies were eligible for and were meta-analyzed, showing significantly higher circulating ANGPTL8 levels in patients with NAFLD or MAFLD (six and one study for the NAFLD or MAFLD diagnosis, respectively) compared to controls (SMD: 0.62, 95% CI: 0.28, 0.97, p < 0.001). This finding is in accord with and updates the 2021 systematic review by Ke et al. (44) which also showed that circulating ANGPTL8 levels in patients with NAFLD were significantly higher compared to those in individuals without a NAFLD diagnosis (SMD: 0.97, 95% CI: 0.77, 1.18). The seemingly higher reported SMD in this 2021 meta-analysis may be attributed, at least partly, to the inclusion of mostly studies published as papers written only in the Chinese language (45–49), whilst it should be also noted that the Hedges’ g is reported as the measure of effect size in our random effects meta-analysis since it corrects for small sample bias, thus providing a more consistent estimate across studies compared to other effect size measures (e.g., the Cohen’s d) (25).

Although the present findings are significant and agree with the previously reported relevant evidence, it should be highlighted that considerable between-study heterogeneity [*I^2^* = 77%; Q = 25.55 (df = 6), p < 0.01] was observed among the included studies. In the sensitivity analysis, removing the Gan et al. study (42), which was the only included study with the diagnosis of MAFLD (and not NAFLD), reduced the overall heterogeneity (*I^2^* reduced to 43% from 77%), while the summary effect remained significant (SMD = 0.50, 95% CI: 0.24, 0.75, p < 0.01). Thus, the outlier study by Gan et al. with the MAFLD diagnosis appears to contribute to the noted heterogeneity. On the contrary, removing the one study identified as ‘Poor’ according to the NIH quality assessment (37) did not have a substantial impact on the overall heterogeneity (*I^2^* = 79%), whilst it slightly increased the overall SMD (SMD: 0.67, 95% CI: 0.29, 1.05, p = 0.006). Moreover, the performed subgroup analyses show diminished heterogeneity (*I^2^* = 0%) between studies which reported using the same Wuhan EIAab Eliza kit for the measurement of circulating ANGPTL8 levels, with a similar SMD (SMD: 0.61, 95% CI: 0.28, 0.94. p < 0.01). Thus, the ANGPTL8 measurement method/kit may represent another variable which contributes to the heterogeneity noted among the studies of the present meta-analysis. Finally, in the subgroup analysis based on method of NAFLD/MAFLD diagnosis, the subgroup with an ultrasound-based diagnosis showed the highest heterogeneity (*I^2^* = 87%), whilst also having the greatest SMD (SMD = 0.71, 95% CI: 0.02, 1.40), suggesting that the method of NAFLD/MAFLD diagnosis maybe also contributing to the documented heterogeneity. However, the overall number of included studies, and accordingly the number of studies in each subgroup of the performed subgroup analyses, is small; hence, attributing the noted heterogeneity to specific factor(s)/variable(s) requires caution.

### 4.1 Potential pathophysiologic links between ANGPTL8 and steatosis/steatohepatitis

ANGPTL8 has emerged as a central regulator of glucose and lipid metabolism, and its dysregulation has been associated with various cardio-metabolic disorders, including obesity, insulin resistance and dyslipidaemia (14, 15). During the course of steatosis/steatohepatitis, the risk of developing glucose intolerance and T2DM increase significantly (50–52). Elevated ANGPTL8 levels in steatosis/steatohepatitis have been hypothesized to represent a compensatory mechanism to mitigate hepatic insulin resistance and hyperglycemia, potentially *via* enhanced β-cell proliferation and insulin secretion (53). In addition, ANGPTL8 inhibits lipoprotein lipase activity, thereby influencing triglyceride metabolism (54). Indeed, a synergistic interaction of ANGPTL8 with ANGPTL3 and ANGPTL4 (under the “ANGPTL3-4-8” model) significantly impacts lipid flux, storage, and triglyceride clearance, leading to elevated serum triglyceride levels (55, 56). Notably, the association of ANGPTL8 with insulin resistance provides insight into its potential underlying mediating role in steatosis/steatohepatitis. ANGPTL8 levels correlate positively with insulin resistance and triglyceride levels which are both key contributors to hepatic steatosis and steatohepatitis progression (36). Furthermore, the relationship between ANGPTL8 and NAFLD appears to weaken when adjusted for insulin resistance, suggesting a mediating role of the latter (40).

In terms of disease severity, our subgroup analysis showed no significant difference in circulating ANGPTL8 levels between NAFLD patients and controls when the corresponding studies reported the NAFLD severity either as mild or as moderate-to-severe (**Figure 5**). However, the number of eligible studies in each of these subgroups is only two, hence more studies are required to clarify the potential association of circulating ANGPTL8 levels to the severity of steatosis/steatohepatitis. Individual studies have reported that ANGPTL8 expression appears to vary significantly depending on the NAFLD stage/severity (38, 39, 43). Indeed, it has been reported that in early NAFLD stages, circulating ANGPTL8 levels may be elevated, potentially serving as a compensatory response to mitigate hepatic insulin resistance and lipid dysregulation (38, 57). Moreover, the studies by Lee et al. (40) and Hong et al. (39) reported increased circulating ANGPTL8 levels in patients with NAFLD, regardless of obesity or diabetes status. This increase may act to promote triglyceride redistribution and β-cell proliferation, enhancing insulin secretion to counteract the metabolic stress (53). However, Sönmez et al. (38) reported significantly lower circulating ANGPTL8 levels in NASH patients compared to NAFLD patients without NASH, while the latter group had significantly higher ANGPTL8 levels than the controls. These were also inversely correlated to histological severity, suggesting that ANGPTL8 downregulation may reflect impaired hepatic adaptive capacity or exhaustion of compensatory mechanisms. Furthermore, the study by Hong et al. (39) reported that circulating ANGPTL8 levels increased by 52% and 48% in patients with moderate-to-severe NAFLD compared to those without or with mild NAFLD, respectively. Similarly, the study by Cengiz et al. (43), which reported lower circulating ANGPTL8 levels in patients with biopsy-proven NAFLD compared to healthy controls, further showed that the lowest levels were in those with significant fibrosis compared to those with mild fibrosis. Mechanistically, this ANGPTL8 reduction during more advanced steatohepatitis may be attributed to progressive hepatic dysfunction with decreased ANGPTL8 synthesis or suppressed ANGPTL8 expression by the local chronic inflammation (38, 56). Elevated ANGPTL8 levels in earlier steatosis/steatohepatitis stages may exacerbate disease progression through inhibitory effects on the lipoprotein lipase activity (38, 56). ANGPTL8 reduces triglyceride clearance and promotes ectopic lipid accumulation, which can contribute to steatosis and its downstream complications (38, 56). Furthermore, the implication of ANGPTL8 in nuclear factor-κB (NF-κB) mediated inflammation and autophagy, may accelerate the transition from steatosis to fibrosis (58). Indeed, chronic hepatic inflammation is the hallmark of steatohepatitis, and data indicate that ANGPTL8 is implicated in the regulation of pro-inflammatory pathways, including NF-κB signaling, which promote fibrosis and cirrhosis (42, 58). Finally, the involvement of ANGPTL8 in lipid and glucose metabolism renders it as potential therapeutic/drug target in the context of steatosis/steatohepatitis, since, via the PI3K/Akt signaling pathway, ANGPTL8 enhances insulin sensitivity and promotes glycogen synthesis, while it inhibits gluconeogenesis (59, 60).

### 4.2 Strengths of the present systematic review and meta-analysis

The present systematic review and meta-analysis has several strengths, including a rigorous study search/identification strategy (i.e., searching for existing eligible studies on NAFLD or MAFLD or MASLD) in key international biomedical databases and a comprehensive quality assessment. Of note, this meta-analysis updates the 2021 meta-analysis by Ke et al. (44), while it includes more studies published in English in international peer-reviewed journals. Herein, we also present a robust meta-analysis, reporting the Hedges’ g as the effect size measure of our random effects model which corrects for small sample bias (25). Thus, the present systematic review and meta-analysis further reduced potential limitations/biases relating to inclusion of small sample studies or a majority of studies published only in Chinese, whilst the main finding agrees with the 2021 meta-analysis by Ke et al. (44). The robustness of the present findings is further reinforced by the performed sensitivity analyses, outlier diagnostics, publication bias analyses, study-level power analyses, and tests for data manipulation. This comprehensive battery of analyses and tests allowed us to scrutinize the data included in the meta-analysis, addressing key issues around data skewness, outliers that excessively influenced the pooled result and contributed disproportionately to between-study heterogeneity, as well as evaluating whether our meta-analysis was based on robust or underpowered studies. Finally, the systematic evaluation of potential publication bias enhances the credibility and generalizability of the reported outcomes.

### 4.3 Limitations of the present systematic review and meta-analysis

Although our meta-analysis includes the largest number of relevant studies published in English to date, the number of available eligible studies is still limited. Thus, the relative paucity of existing eligible studies may impact on the robustness of the present findings. Additionally, the small sample size and quality/bias issues of several of the eligible studies, as well as the documented substantial heterogeneity, may also affect the strength of the meta-analyzed evidence. The different methods utilized by the eligible studies for the diagnosis of NAFLD and their inherent limitations (e.g., concurrent inflammation, congestion, cholestasis, and alcohol intake are potential contributors to the heterogeneity in ultrasound-based diagnosis of NAFLD) may additionally limit the strength of the meta-analyzed evidence. Furthermore, due to lack of reporting data on all relevant metabolic comorbidities or the way that such data are reported in the papers of the included studies it is not clear if all the studies that applied the NAFLD diagnosis can be appropriately compared to studies applying the MAFLD and/or MASLD diagnostic criteria. Moreover, the available data are derived from observational/cross-sectional studies, and, thus, the ability to establish causal relationships between circulating ANGPTL8 and the development/etiology or progression of NAFLD/MAFLD is intrinsically limited. Of note, two of the included studies (41, 42) reported a case-control study design in the title and/or methods of the corresponding papers, although we discerned that these were cross-sectional; in the present systematic review, we analyzed these two studies based on the latter rather than on what was reported as a case-control design in the corresponding published article. In addition, based on the applied eligibility criteria, this systematic review included only studies in adults, and thus, the present findings cannot be generalized to non-adult patients with steatosis/steatohepatitis and those with genetic syndromes linked to severe obesity and steatosis/steatohepatitis. Finally, according to the registered PROSPERO protocol, the present systematic review searched exclusively for English-language published papers; hence, potentially eligible non-English papers/studies, such as studies published as papers written only in the Chinese language (45–49), were excluded *a priori*.

## 5 Conclusion

This systematic review and meta-analysis present the most up-to-date and comprehensive synthesis of current evidence on the association of circulating ANGPTL8 to steatosis/steatohepatitis, having searched for eligible studies under all relevant terms (i.e., NAFLD, MAFLD and MASLD). The present findings indicate that patients with NAFLD or MAFLD (no eligible study with a MASLD diagnosis was identified) exhibit higher circulating ANGPTL8 levels compared to controls, highlighting the potential of circulating ANGPTL8 as an additional novel biomarker for steatosis/steatohepatitis. Given the clinical need for novel biomarkers to improve the non-invasive screening and diagnostic approaches for steatosis/steatohepatitis, large and prospective studies are required to further explore this potential association of circulating ANGPTL8 to various stages of steatosis/steatohepatitis, and to elucidate its temporal/causal direction, particularly under the new MASLD diagnosis/term. Advancing our understanding of the role of ANGPTL8 in the context of this chronic liver disease may also pave the way for innovative therapeutic approaches for patients with steatosis/steatohepatitis which would target ANGPTL8-related pathways.

## Data Availability

The original contributions presented in the study are included in the article/**Supplementary Material**. Further inquiries can be directed to the corresponding authors.
